# Exploring the construction and infiltration strategies of social bots in sina microblog

**DOI:** 10.1038/s41598-020-76814-8

**Published:** 2020-11-13

**Authors:** Wenxian Wang, Xingshu Chen, Shuyu Jiang, Haizhou Wang, Mingyong Yin, Peiming Wang

**Affiliations:** 1 College of Cybersecurity, Sichuan University, Chengdu, China; 2 Cybersecurity Research Institute, Sichuan University, Chengdu, China; 3 College of Computer Science, Sichuan University, Chengdu, China

**Keywords:** Computer science, Information technology, Information theory and computation, Software

## Abstract

Nowadays, millions of people use Online Social Networks (OSNs) like Twitter, Facebook and Sina Microblog, to express opinions on current events. The widespread use of these OSNs has also led to the emergence of social bots. What is more, the existence of social bots is so powerful that some of them can turn into influential users. In this paper, we studied the automated construction technology and infiltration strategies of social bots in Sina Microblog, aiming at building friendly and influential social bots to resist malicious interpretations. Firstly, we studied the critical technology of Sina Microblog data collection, which indicates that the defense mechanism of that is vulnerable. Then, we constructed 96 social bots in Sina Microblog and researched the influence of different infiltration strategies, like different attribute settings and various types of interactions. Finally, our social bots gained 5546 followers in the 42-day infiltration period with a 100% survival rate. The results show that the infiltration strategies we proposed are effective and can help social bots escape detection of Sina Microblog defense mechanism as well. The study in this paper sounds an alarm for Sina Microblog defense mechanism and provides a valuable reference for social bots detection.

## Introduction

With development of information technology and the popularity of the Internet, more and more people express their personal views and opinions through the Internet. The Internet has become the main way for people to release and obtain information. It is reported that, by April 2019, Global Internet users grew by 8.6% over the past twelve months, with almost 3.5 billion people using social media^1^. As one of the most popular online social media, Sina Microblog had 486 million active users in June 2019 and average daily users of 211 million^2^. It has become an important channel for the masses to obtain information and express their views and attitudes on current hot issues. However, due to its openness and freedom, some irrational users or spammers will release various kinds of harmful information such as rumor^3, 4^, hate speech^5, 6^ and fake news^7^ on the platform. They usually deliberately interpret or comment on certain events maliciously, guiding and inciting the negative emotions of other users. This would cause adverse effects on enterprises, institutions and even government departments. Therefore, it is of great significance to dilute harmful information spreading and guide public opinion in a positive way when a major emergency occurs^8^.

According to the research report of Oxford Internet Institute^9^, the influence of the social bots on mainstream OSNs in the United States, Russia, Germany, Canada, China and other countries can not be underestimated. Social bots, as a computer program, can control social accounts, automatically post tweets on social platforms and use relevant technologies such as artificial intelligence to mimic and interact with human users^10^. At present, OSNs such as Twitter and Facebook have found more and more social bots, which has profoundly affected many fields such as economy, politics and people’s social life^11–14^. And it has been proved that social bots were largely responsible for the massive spread of misinformation, which posed a major threat to democracies^15, 16^. Bessi et al. even^12^ found that social bots were very active in the online political discussion of the 2016 USA presidential election and posted nearly 3.8 million tweets accounting for one-fifth of the total. Their experiments suggested that both the Hillary Clinton and Trump teams have used social bots to conduct political propaganda on Twitter and attack the opponent. The same phenomenon of political social bots was also discovered by Woolley et al.^17^, and they conducted an in-depth analysis of these bots’ media articles. Hence the analysis of social bots can help control the spread of harmful information.

As one of the largest and most popular OSNs in the world, Sina Microblog allows some irrational users to perform malicious behaviors due to its high openness. These malicious behaviors usually include: (a) confusing international public opinion^11, 14, 18^; (b) spreading negative emotions^19, 20^, which could cause social panic; (c) commercial misconduct^21^, like posting fraudulent links, malicious slander of the company, product or public figure. These could cause serious impacts on the normal operation of society, people’s daily life, and business activities of enterprises.

The current researches on social bots mainly focus on the detection^20, 22–27^, while research about the construction and infiltration strategies of social bots was rarely. The research about social bot detection often requires irrational users to do malicious behaviors causing losses to OSN before they can be detected. It is a passive defense, and it is difficult to form an effective and timely resistance sometimes. Therefore, we eager to seek a more effective method to research possible bot construction and infiltration strategies of social bots. So that we can actively find the existing shortcomings to formulate a response plan in time. At the same time, these constructed social bots can also create a harmonious social network atmosphere by posting positive comments. Besides, it can provide a reference for the social bots detections as well. The social bots in Facebook or Twitter have been studied by some researchers^28–30^. However, due to differences of users, social network structure, active time, regulatory requirements and other aspects, the strategies to construct social bots of Sina Microblog need to be different.

For these reasons, this paper constructed batches of social bots in Sina Microblog and studied how various characteristics of social bots affect their infiltration performance.

In summary, the contributions of this paper are as follows:

- We studied the critical technology of data collection in Sina Microblog and took advantage of the deficiencies of its defense system to build multi-strategy social bots, indicating the vulnerability of Sina Microblog defense mechanism. In the experimental stage, we nurtured 96 social bots with a survival rate of $100\%$ using reverse engineering to collect data, deep learning to generate positive comments for responding to regulatory requests of building healthy cyberspace, and other technologies to set profiles and activities. Moreover, these social bots total gained 5546 followers within the 42-day infiltration.
- Based on the constructed social bots, this paper evaluates the infiltration performance of social bots from five aspects: gender, profile photo, activity level, following strategies and posting strategies. The results showed that if a social bot wants to gain more followers in a short time, it was more effective to: (a) set the gender and profile photo to female; (b) act in a high activity level (the interval between two consecutive activities is between 20 and 150 min at random); (c) follow users with a specific set of targets (like following users with the same interest) instead of following them randomly.
- Then, this paper further researched which interaction behavior is more successful in expanding the infiltration scale through two comparative experiments. The results showed that following followers’ followers is the quickest way to gain followers and enhance influence. This also indicates that homophily can make social bots in Sina Micrbolg more influential.

We are aware that the research in this paper may pose potential ethical problems and the proper use of social bots will be necessary for guiding applications. There is a risk that this research could be used to manipulate social bot armies for public opinion attacks or political manipulation. It should be noted that our starting point is to build positive and interesting social bots to help create a harmonious network environment and understand the infiltration strategies of social bots. Thus, in our experiments, all social bots were set to only concern about the games, technologies and life news, avoiding sensitive topics such as politics and the military. Meanwhile, the contents posted by social bots were set to be positive or neutral, avoiding generating negative speech.

## Related work

From the beginning of OSNs to the present, there has been a lot of research devoted to the creation and infiltration of social bots in OSNs. These works can be categorized into two main types: (1) researching social bots themselves, such as their construction, infiltration strategies and the ability to collect personal information; (2) analyzing the characteristics of infiltrated groups.

Up to now, most of the research on the construction and infiltration of social bots was about Twitter and Facebook OSNs. In the early research on Twitter OSN, the Realboy^31^ project called Twitter APIs to realize the functions of automatic posting and commenting tweets, automatic following of specific users, etc. This project laid the foundation for the subsequent research on Twitter social bots. In subsequent studies, Freitas et al.^29^ constructed 120 social bots with different attributes based on the Realboy^31^ and studied four infiltration strategies (gender, activity level, tweet generating strategy and target users) which intuitively affected how successful social bots were in infiltrating Twitter OSN. Their bots continued to be active on Twitter for 30 days and 69% of the bots were undetected by Twitter at the end of the experiment. Moghaddam et al.^28^ also studied similar attributes and infiltration strategies with 128 social bots in two 40-day experiment cycles. However, they further researched how the homophily affected social bots’ influence and found that the common characteristics and similarity indeed would increase the probability of being followed by other users. These studies revealed the vulnerability of Twitter to large-scale social bot infiltrating. Similarly, Zhang et al.^32^ had built large-scale social bots as well. Three social bots networks were constructed by them in Twitter OSN and each social bot network consisted of 100 social bots. The social bots in each network were divided into spam publishers and forwarders. Then spam publishers posted malicious content and forwarder retweeted these tweets after a short time. They found that Twitter’s anti-spam system only blocks spammers and did nothing about forwarders.

Unlike previous studies, Messias et al.^33^ using two social bots demonstrated even simple strategies can make social bots influential, although this was only a small “proof of concept”. They deployed two social bots in Twitter OSN and kept social bots tweeting about hot topics and following users in 90 days. These social bots obtained a high Klout score and a certain number of followers. Shafahi et al.^34^ deployed eight social bots in Twitter OSN, each of which was related to a specific topic, to study the effects of tweet strategy, gender and following strategy on the infiltration performance of social bots. During the 4-week infiltration, all bots attracted more than 410 Twitter users from 48 different countries to their phishing sites, of which at least 33 users visited their phishing sites from the company network. They found that it is possible to lead employees to a website by using shortened links in tweets. Their work suggested that phishing through social bots could pose a serious threat to companies. Savvopoulos et al.^35^ used automatic conversation technology on the basis of literature^36^ to study the role of automatic chatting in social bots’ infiltration on Twitter OSN. Their study found that the chat function can increase Klout and the number of followers by about 24% and 123% respectively.

Compared with Twitter OSN, the infiltration on the Facebook OSN pays more attention to the infiltration of specific organizations and the ability to collect personal information. Huber et al.^37^ deployed the ASE social bot in Facebook OSN and used it to carry out automated social engineering attacks. In this research, the ability of social bots to collect user information and the results of their Turing tests were examined. Their research showed the technical feasibility of automated social engineering attacks. Elishar et al.^38^ used two female social bots successfully infiltrate the two institutions using and discovered up to 18.29% more informal organizational links and up to 13.55% more employees and compared with public ones. This further proved the vulnerability and information leakage of Facebook OSN. Boshmaf et al.^39, 40^collected more than 250 GB of Facebook users’ information through maintaining friendships with human users. They adopting the traditional web-based botnet built a Socialbot Network (SbN). This SbNA continued to run for about eight weeks with an 80% infiltration rate. Their work proved the feasibility of privacy breach by exploiting social bots. Elyashar et al.^30^ studied the infiltration of social bots in organizations related to computer technology, whose employees should theoretically be more security-conscious. However, their experiments showed that the infiltration of computer-related workers also had a high success rate and found that the more mutual friends the user had, the more likely he or she was to accept a friend request from a social bot.

On the other hand, the characteristics of the infiltrated groups can help understand the principle behind infiltration strategy and infiltrate OSNs more sunccessfully. Usually, women were more likely to be deceived by phishing sites than men and the age group between 18 and 25 was more susceptible than other age groups^41^. Wagner et al.^42^ studied the characteristics of user groups that were easily infiltrated by social bots, including 70 language features, three network-related features and 13 behavioral features. The authors found that susceptible users tended to have larger social relationship graphs. Such users tended to use Twitter as a conversation platform and use more social vocabulary, showing more emotion than non-susceptible users. Wald et al.^43^ studied 610 real users who interacted with social bots. Six classifiers constructed by them to determine which features made users most likely to interact with social bots and experiments showed that users with high Klout scores and large number of followers were more likely to interact with bots. Heartfield et al.^44^ found that for automated social attacks, users with computer security awareness and more familiarity with the use of specific social platforms were less likely to be deceived by bots. Fazil et al.^45^ deployed 98 social bots on Twitter and all social bots were allocated to different countries according to the proportion of Twitter users in different countries. From this experiment, they found social bots’ profiles belonging to India were successful in cheating users, while Indonesian social bots were least infiltrative. Subsequently, Fazil et al.^46^ randomly selected 749 users from all the collected Twitter user information and divided them into active users, reactive users and inactive users based on their interaction with social bots. The results showed that active and reactive users keep on frequently updating their tweets containing advertising related contents. They also used feature ranking algorithms to analyze features’ discriminative power and found that the following rate and follower rate were the most dominating features.

As mentioned above, most of the research on the construction and penetration of social bots was about Twitter and Facebook OSNs, while the related research about Sina Microblog OSN^47, 48^ was little. Although Liu et al.^48^ deployed a social bot on Sina Microblog to help users filter out useless messages, their work was more inclined to build an intelligent “information agent”, rather than to study the batch construction and infiltration strategies of social bots. Other related works about Sina Microblog OSN were paid more attention to the construction and detection of malicious botnets^49–51^. However, Sina Microlog OSN is often flooded with malicious speech and some collective personal attacks will lead to public opinion accidents. Therefore, this paper studied the large-scale automatic construction method and infiltration strategies of social bots in Sina Microblog OSN, hoping that these benign social bots can be used to introduce positive guidance to malicious public opinions.

## The framework

**Figure 1:**
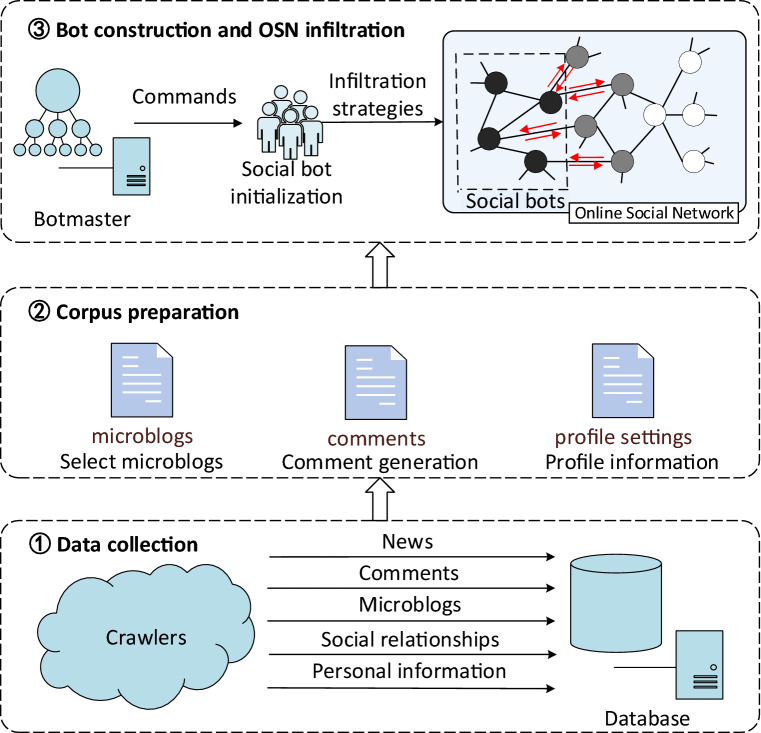
The framework. On the right of the 3rd subgraph, each node in the OSN represents a user and edges between nodes stand for social connections. The black nodes represent social bots and infiltrated users are marked in gray. The red directed arrows represent social interactions.

Figure 1 shows the framework for building social bots and infiltrating Sina Microblog OSN. As shown in Fig. 1, it mainly includes three parts: data collection, corpus preparation, and social bot construction and OSN infiltration. Firstly, a batch of crawlers is constructed to crawl personal information, social relationships, microblogs and comments in Sina Microblog and news to form an information database. Then, based on this information database, the corpus of profile settings, comments and microblogs to be published are well prepared using pattern matching, deep learning and other technologies. Finally, a social bot control software, which is called *Botmaster* through commands and this corpus to build social bots, and control them to perform activities according to the preset infiltration strategies.

## Bot construction

In this section, we first initialized the profiles of social bots in Sina Microblog. Then we divided daily actions of social bots into two types: (1) *Social-Interaction actions (SI actions)* and (2) *Social-Structure actions (SS actions)* and a set of commands based on this were created to manipulate social bots. All the data collected by crawlers was stored in the database. Then, we set the daily actions that each social bot needs to perform as commands. The *Botmaster* reads these commands and transmits them to bots. As shown on the right of the 3rd subgraph of Fig. 1, social bots infiltrate OSN through interacting with other users.

For the experiments, we totally created 96 social bots, using 9 cloud servers with independent public IP addresses. As is shown in Table 1, 10–12 social bots were assigned on each server for infiltration in 6 weeks.

**Table 1:** Experimental environment.

Setting	Value
Number of cloud servers	9
Server location	BeiJing, GuangZhou, Chengdu etc.
Size of server memory	2G
Bandwidth of sever	1M
Number of social bots on each server	10–12
Number of social bots	96
Infiltration period	6 weeks

### Sina microblog data collection

Data collection is the basis of building social bots. However, obtaining data through official APIs of Sina Microblog is fairly restricted, because API request frequency is limited by IP and account. Fortunately, we cracked the password encryption process and the simulated login process in Sina Microblog using reverse engineering and then developed crawlers based on these to collect data automatically^52^.

**Figure 2:**
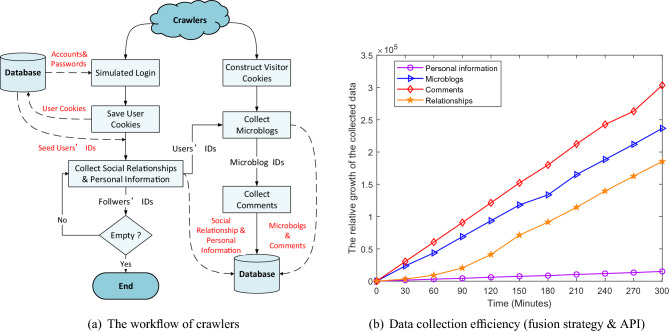
(**a**) The workflow of crawlers; (**b**) the comparison of data collection efficiency using the fusion strategy and APIs under the same condition.

Since the infiltration of Sina Microblog OSN needs *users’ social relationships, users’ personal information, microblogs* and *comments* as the basis, these four types of information would be collected by our crawlers. We adopt a fusion strategy which combines the simulated login and visitor cookies to crawl Sina Microblog data and use the concurrent adaptive strategy^52^ to control concurrent threads and cookies. The workflow of our crawlers is illustrated in Fig. 2a. Firstly, crawlers conduct simulated login and construct visitor cookies. Then, login cookies and visitor cookies are respectively saved into different cookie queues. After that, crawlers crawl the social relationships and personal information of seed users with login cookies and users’ IDs got from seed users’ social relationships will be stored as the new seed users for the next collection. Meanwhile, these users’ microblogs and comments will be collected by crawlers with visitor cookies. Finally, crawlers will repeat the steps above according to the breadth-first strategy^53^ until there are no followers. Figure 2b compares data collection efficiency using the fusion strategy and official APIs. It can be seen that using the fusion strategy to collect data is much faster than using official APIs.

### Comment generation

**Figure 3:**
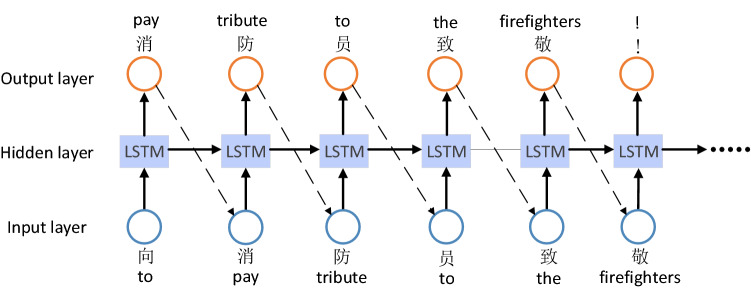
Char-RNN.

To avoid bringing negative effects to Sina Microblog OSN, all comments posted by social bots should be positive or neutral. Therefore, we used the LSTM with word embeddings^54^ as the sentiment classifier to distinguish between positive and negative comments, and then used Char-RNN^55^ as the text generation model to generate positive comments. After training the sentiment classifier, we used it to filter out positive comments which were used to train the text generation model. Char-RNN model is shown in Fig. 3. The model consists of two LSTM layers and a dense layer, choosing *Adam* as the optimizer and *categorical_crossentropy* as the loss function.

**Table 2:** The comments generated under the different temperature parameter *T* for a chemical plant explosion event.

Temperature	Generated comments
0.2	
	Fire fighters must return safely
0.5	
	Hope the fire fighters can return safely!
0.8	
	Must pay attention to safety and wise everyone a safe return
1.0	
	Brother fireman has worked hard, please be safe!
1.2	
	I remembered that there were newborn children rescued by fire fighters, they must be sage too

Here we use the *softmax* function as the activation function to generate comments. At the same time, we introduced the temperature parameter *T* of the softmax function^56^ to control the randomness in the sampling process, so as to generate more creative comments. The probability distribution of the *i*th character $y_{i}$ is calculated as Eq. (1) where *z* is the output of the previous network layer, *C* is the dimension of *z* and *T* is the temperature parameter. The smaller *T* is, the more repetitive and the less diverse generated comments are. Similarly, when *T* becomes larger, the novelty of generated comments will be higher, but at the same time, grammatical errors and context-independent situations are more likely to occur. Table 2 shows the comments generated at different temperatures for a chemical plant explosion event.

1 $$\begin{aligned} P(y_{i}|(y_{1},\ldots ,y_{i-1}))=S(z_{i})= \frac{e^{z_{i}/T}}{\sum _{j=1}^{C}e^{z_{j}/T}}, i=1,2,\ldots ,C. \end{aligned}$$

### Profile settings of social bots

In order to make social bots look similar to human users and have high credibility, their profiles need to be personalized. User profiles in Sina Microblog include *basic information*, *contact information*, *career information* and *education information*. These profile attributes would be set for social bots as well. In the following content, we describe how to set up these four types of information. Specific profile attributes are shown in Table 3.

**Table 3:** Attributes of profile.

Type of attributes	Attributes
Basic information	Nickname & Gender&
	Real name & Birth date&
	Location & Hobbies & Others
Contact information	QQ & E-mail
Career information	Company
Education information	University & Enrollment year

#### Basic information

Personal information like nickname, real name, gender, birth date and location are all included in basic information.

- *Nickname&Gender:* Nickname in Sina Microblog is the identifier of users and it’s unrepeatable. So, in order to make social bots appear more realistic, we pre-fetched 30,000 users’ information, including nicknames and genders, in Zhihu^57^ and NetEase Cloud Music^58^ as alternative materials and then used the Sina Microblog related interface (https://account.weibo.com/set/aj5/userinfo/checknickname) to query whether the collected nicknames were allowed to register. It is noted that a simulated login is required before the query. If the request returns {code:100000}, the nickname is available, otherwise, it is unavailable. After filtering out users with duplicate nicknames, other users were divided into two groups by gender and respectively ranked by the number of followers. At last, we chose the nickname and corresponding gender of the top 48 users in each group as our social bots’.
- *Real name:* The real name in social bot’s profile was generated according to gender by Faker^59^, a Python open-source library.
- *Birth date:* The birth year of social bot was randomly set one year between 1980 and 2000 and the birth month was randomly set from January to December.
- *Location:* The location was set to the location of the corresponding cloud server.
- *Hobbies:* In the experimental stage, all social bots were divided into three groups. Social bots in different groups were interested in different topics, namely technology, news and games. When social bots made an interest selection, they would choose hobbies related to their topic as well. In the following researches, social bots’ occupations, microblogs they post and target users they infiltrate would all revolve around this hobby.
- *Others:* Sexual reference of our social bots was defined as heterosexuality and blood type was randomly chosen from A, B, AB and O.

#### Contact information

Contact information includes QQ number and E-mail. The QQ number was a randomly generated integer consisting of seven to ten digits. The E-mail was set according to the social bot’s real name. In this paper, the real name was converted to Chinese phonics or English as the first part of an E-mail and the E-mail suffix was randomly created through *Faker*. For example, if a social bot’s real name is “Li Ming” and the E-mail suffix created by *Faker* is “@hotmail.com”, then his E-mail is *liming@hotmail.com*.

#### Career information

We also used Faker to generate work companies in this subsection and most of them were media companies and technology companies considering the hobbies and target users.

#### Education information

Education Information includes university and enrollment year. Considering that the locations of most users in Sina Microblog are the provinces where their universities are located, we randomly chose one university in the province that we set in (1) as the university of the social bot. The enrollment year would be set to the year when social bots were between 17 and 20 years old. On the other way, if the social bot’s age was smaller than 17, the education information settings would be skipped.

After the profile setting of each bot is completed, 4–8 social bots were randomly assigned as the initial followers to each social bot to make our social bots like a human user when infiltrating Sina Microblog. Then at least 10 microblogs were posted continuously in 5 days by each social bot before the start of the infiltration. After that, social bots would act according to the infiltration strategies in “Infiltration strategies of social bots”.

### Activity settings of social bots

Since the goal of our social bots is to infiltrate the OSN and gain influence, it is necessary that they make interactions with other users in OSN. To this end, we defined two types of daily actions that social bots need to perform. These two types are: (a) *Social-Interaction actions (SI actions)* that are used to post and read microblogs; (b) *Social-Structure actions (SS actions)* that are used to alter the OSN structure. Considering that the official APIs of Sina Microblog have many restrictions and are not scalable, all operations in this paper are implemented through sending HTTP packages in a simulated login state. Specific actions of these two types and their definitions are shown in Table 4.

**Table 4:** Definitions of social Bots’ daily actions.

Action	Action type	Action description
Follow	SS action	Follow a user
Unfollow	SS action	Unfollow a user
post_text	SI action	Post a text microblog
post_img_text	SI action	Post a image-text microblog
forward	SI action	Forward a microblog
comment	SI action	Comment on a microblog
like_text	SI action	Give a like to a microblog
like_comment	SI action	Give a like to a comment
reply_comment	SI action	Reply other’s comment
send_msg	SI action	Send a message to a user

Through the execution of SI actions and SS actions, our social bots would establish connections with other users and form their social network to affect the entire OSN. We defined two types of commands to manipulate social bots to perform daily actions: *atomic commands* and *combined commands*. Atomic commands are shown in Table 4 and combined commands consist of multiple atomic commands. After each atomic command is executed, our social bots would sleep for a random short period of several seconds, simulating the network delay and the action interval of human users. The format of both commands is key-value pair: *{“task_id”: tid, “bot_id”: bid, “callback”: action, “args”: args, “prepare_time”: timestamp}*, where *tid* represents the task ID and *bid* represents the social bot ID, *action* indicates the action name. If it is a combined command, the *“callback”* is empty, *args* represents the parameter required to execute corresponding commands, on the other hand if it is an atomic command, the value of *“callback”* is an action name. For example, a combined command: *{“task_id”: 1, “bot_id”: 1, “callback”: “”, “args”: {{“callback”: “follow”, “args”: 6768536764, “fly sound watch drama” }, {“callback”: “follow”, “args”: 5257481279, “the small white rice”}}, “prepare_time”: 1554789558}*, indicates that the social bot with ID 1 follows users with ID 6768536764 and ID 5257481279 when the Unix timestamp is 1554789558.

In addition, in order to increase the credibility of social bots to Sina Microblog Detection System, each social bot used a fixed User-Agent when performing various actions.

## Infiltration strategies of social bots

In this section, different infiltration strategies were proposed and experimented to find the best infiltration strategy.

**Figure 4:**
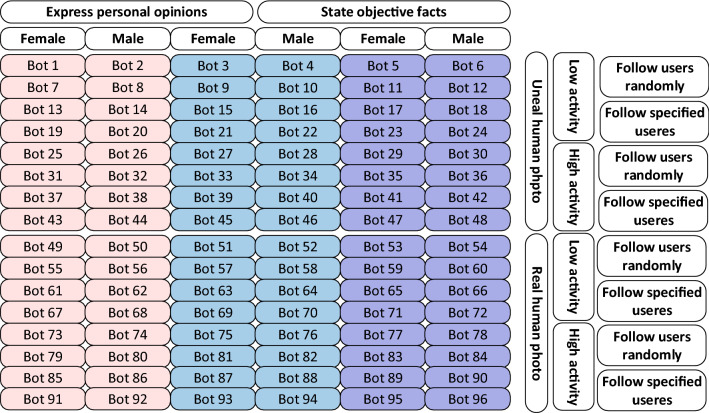
Distribution of strategies adopted to social bots. The pink, blue and purple grids represent social bots in different topic groups, namely technology, news and games. And the numbers in these grids represent social bot IDs. The white grids represent different strategies, and social bots in corresponding columns or rows perform activities with these strategies.

There are quantities of factors that could potentially influence how other users view a user-account in Sina Microblog. Since analyzing the impact of all possible factors is almost impossible, we set up five strategies to measure the intuitively most important factors that may determine how successful a social bot is in infiltrating Sina Microblog OSN. These five factors are: *(a) gender; (b) the type of profile photo; (c) the activity level; (d) the following strategy*; *(e) the posting strategy*. In the experiments, all social bots were equally divided into three groups according to the topics they were interested in, which are technology, news and games respectively. Each group are assigned relevant attributes as shown in Table 5 and Fig. 4 details the distribution of strategies adopted by each social bot.

**Table 5:** Infiltration strategies of social bots. The percentage in table represents the ratio of the number of social bots assigned to this strategy to the total number of social bots.

Factor	Strategy (%)
Gender	Female (50%)
	Male (50%)
Activity level	High (50%)
	Low (50%)
Profile photo	Real human photo (50%)
	Unreal human photo (50%)
Following strategy	Follow specified users (50%)
	Follow users randomly (50%)
Posting strategy	Express personal opinions (50%)
	State objective facts (50%)

### Gender

In order to verify the influence of gender on the infiltration, half of the 96 social bots created in this experiment are designated as males and the other half are designated as females. Setting the social bots’ gender involves the gender setting in profile and using a proper name and profile photo.

### Activity level

The activity level is the frequency of a social bot performing daily actions. We set two activity levels to study whether the activity level of social bots is related to the acquisition of social influence. These two activity levels are:

- *High activity:* For highly active social bots, the time interval between two consecutive activities is randomly between 20 and 150 min;
- *Low activity:* For low-activity social bots, the interval between two consecutive activities is randomly between 60 and 300 min. In this paper, half of social bots are set as high activity and the other half are set as low activity.

Although more active social bots are more likely to get new followers, they are also more likely to be detected as spammers by Sina Microblog defense mechanism. It is therefore important to set the active time and frequency ingeniously. In this article, all social bots will sleep between 0:00 am and 8:00 am to simulate the sleep schedule of human users. If an IP frequently sends requests to the server, the social bots on this IP will also be in danger of being detected as spammers. So we further constrains the active conditions of social bots: if the time of last action made by social bots on the same IP is less than 1 min from the time of the next action, the next action will be delayed by 30–120 s and then executed.

### Profile photo

Using a photo of a real person as an avatar in Sina Microblog is usually seem more convincing, so we decided to find whether to use a real human photo as the profile photo may affect a social bot’s social influence. Half of male social bots and half of female social bots use real human pictures as the profile photos and others use unreal human pictures such as landscapes, cartoons and animals.

What should be noted is that when the social bot uploads a profile photo, it is necessary to specify the clipping mode of the profile photo, which is mainly determined by three parameters: *ax*,*ay* and *aw*. If these parameters are inappropriately set, the uploading will fail. The *ax* and *ay* represent the starting position of the upper left corner of the profile photo and *aw* represents the diameter of the clipped profile photo. We set both *ax* and *ay* to 0 in this paper and then *aw* is calculated as shown in Eq. (2)

2 $$\begin{aligned} aw=min(height,width,900) \end{aligned}$$

*height* represents the height of the profile photo, *width* represents the width of the profile photo and *900* is a relatively large and stable value that we have concluded after a number of tests. The *aw* parameter takes the minimum of the three. In addition, the image needs to be encoded in base64 before uploading in Sina Microblog, which is the same for the *post_img_text* action.

### Following strategy

Another potential factor affecting the infiltration performance of social bots is the collection of target social users. People with similar interests are often more attractive to each other. Therefore, we set up the two following strategies to explore whether it is true for users in Sina Microblog OSN:

- *The half of social bots only follow users who are interested in a common topic;*
- *The others randomly follow other users*.

In addition, in order to prevent our social bots from establishing contact with other fake users or marketing users, the social bots will use these tricks to filter target users when doing *follow* action: (a) the target user should have posted an original microblog or forwarded others’ microblog at least in last month; (b) the number of followers of the target user should be larger than 20; (c) the proportion of a target user’s follower and users followed by him should be no more than 0.01; (d) the target users’ profiles should be complete, at least including profile photo, gender, nickname and introduction.

### Posting strategy

The microblogs posted in Sina Microblog is roughly divided into two types: personal opinions and objective facts. Compared with the description of objective facts, personal opinions are more subjective and more likely to affect users with the same feelings. To verify whether this applies to Sina Microblog OSN as well, we adopted the following two strategies to explore:

(a) *Repost or forward microblogs posted by other users who are interested in the same topic.*

The selected microblogs come from the latest microblogs that were posted or forwarded by individual authenticated users (Sina Microblog authenticated users, which are also called V users (V is the first letter of VIP), are real-name authenticated and unique. They are divided into yellow V users and blue V users. The yellow V user is certified by individuals, like actors, singers, writers, etc. And the blue V user is certified by enterprises or state organs, like companies, studios, universities, etc.). These users’ microblogs are chosen because the authenticated users can avoid spammers and these users’ words have certain influence and subjectivity.

When reposting a microblog, we adopt synonym replacement to re-edit the microblogs. Firstly, the microblog text is segmented by *jieba*^60^ word segmentation tool and then words are replaced by synonyms using *HIT-CIR Tongyici Cilin (Extended)*^61^. When forwarding a microblog, a positive comment on the forwarded microblog is added along with it. During the experiments, these two behaviors are performed with equal probability.

(b) *Post high-quality news of relevant topics gotten from authoritative official media.*

The news reported by authoritative official medias is authentic and objective in theory, so we choose these as microblogs that describe objective facts to post.

## Can social bots infiltrate sina microblog ?

**Figure 5:**
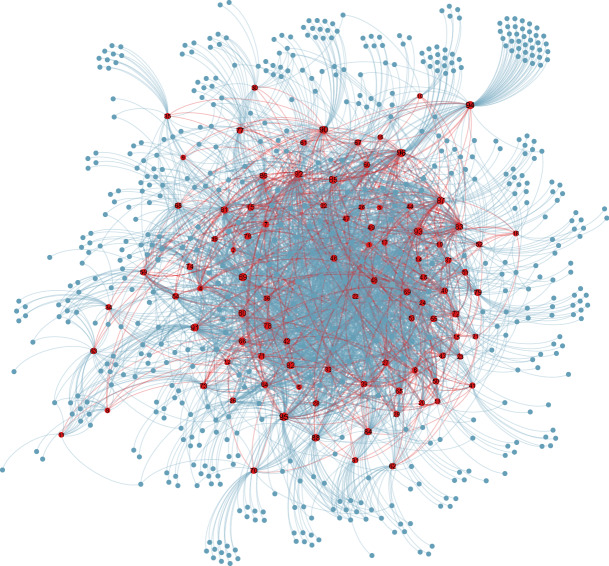
Network topology of the OSN consisting of social bots and their followers, drawn by *Gephi*^62^. The red dots represent social bots and the blue dots represent their followers. The number on the red dot represents the ranking of social bots according to the number of their followers. The connections between social bots are shown in red edges and the connections between social bots and human users are shown in blue edges.

Before analyzing the impact of different infiltration strategies on infiltration effects, it is necessary to investigate whether and to what extent, social bots can infiltrate the Sina Microblog OSN. To this end, our social bots need to achieve the following two goals: (a) evading detection by Sina Microblog defense mechanism which regularly detect malicious crawlers and spammers; (b) gaining a certain popularity and influence in Sina Microblog.

In this section, we analyse how our social bots accomplish the above two goals.

### Social bots can evade sina microblog defense mechanism

First of all, our social bots can avoid the spam account detection mechanism of Sina Microblog. Using the profile settings, activity settings and infiltration strategies in "Bot construction", the 96 social bots were continuously active for 6 weeks in Sina Microblog with a 100% survival rate. We applied *Gephi*^62^ as a visualization tool to draw the social network composed of social bots and their followers, as shown in Fig. 5. It can be seen that although not all social bots directly interact with each other, they have common followers connecting them to form a small and dense OSN. This is very conducive to information dissemination and public opinion guiding because microblogs posted by social bots will be quickly and repeatedly disseminated in this small but dense OSN.

To sum up, all these above indicate infiltration strategies in this paper are reasonable and effective and our social bots can evade the detection of Sina Microblog defense mechanism.

### Social bots can infiltrate sina microblog successfully

**Figure 6:**
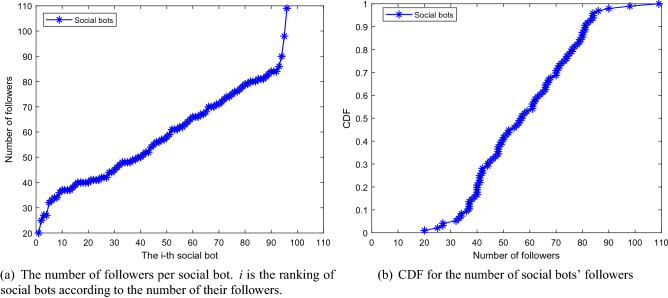
Number of followers.

Secondly, we need to check to what extent our social bots can infiltrate the Sina Microblog OSN. As reported in^63, 64^, the number of followers represents the infiltration scale and user’s popularity, so we use this as the main evaluation indicator. During the 42-day infiltration period, the 96 social bots created in this article gained 5546 followers. Social bots are ranked according to the number of their followers and then the number of followers at the end of experiments for each social bot are is shown in Fig. 6a. Figure 6b shows the cumulative distribution of the number of followers. It can be seen that social bots have obtained a number of followers ranging from 20 to 110. And it is clear that within just 42-day, all social bots had more than 20 followers and 50% of social bots acquired more than 50 followers which is the average number of human users’ followers.

**Figure 7:**
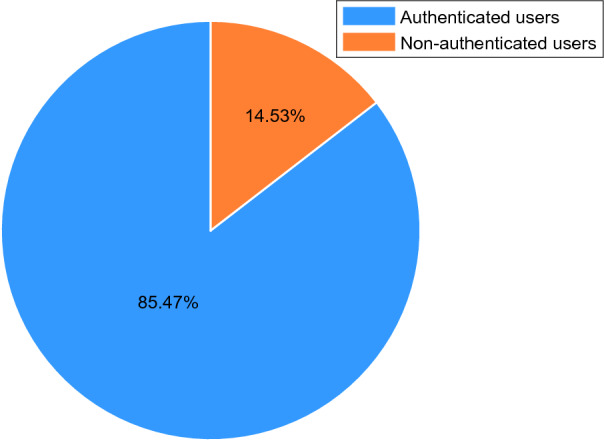
Proportion of authenticated users and non-authenticated users in followers.

Figure 7 shows the proportion of non-authenticated users and authenticated users among all social bots’ followers. The percentage of authenticated follwers has reached 14.53%. What’s more, among all the followers social bots got, 89 authenticated ones had more than 10,000 followers, which means one microblog posted by a social bot has the possibility to be seen by 890,000 users. In addition, during the infiltration process of the experiment, they also received 951 interactions, of which 60.46% were likes, 38.60% were comments and 0.74% were forwarding. This indicates that our social bots have successfully infiltrated Sina Microblog OSN and gained a certain influence.

## Evaluating infiltration strategies

Since the main purpose of this article is to successfully create social bots and expand theirs influence in Sina Microblog OSN, there should be an indicator to measure influence. Considering that the number of followers owned by users represents the popularity of them and the more popular users are, the more likely their microblogs will be recognized and spread by others. Hence we use **the number of social bots’ valid followers**, as the measure of theirs influence. Section already showed our social bots can successfully infiltrate Sina Microblog. In this section, we further studied the efficiency of five infiltration strategies and the infiltration performance of different interaction behaviors through two experimental phases.

We divided the entire experimental process into two phases. In the first phase, we made social bots act per the strategies in “Infiltration strategies of social bots” for 4 weeks. Then, the efficiency of five infiltration strategies was studied from “Gender to Posting strategy”. Furthermore, we quantified the followers of social bots from the perspective of influence, as shown in Section Quantifying the influence of followers. In the second phase, we selected several social bots from the ones in the first phase to form 4 groups. Then we made one group only performed one interaction behavior for 2 weeks in Phase 2. The infiltration performance of different interaction behaviors was compared in “Performance analysis of Interactive actions”.

In order to make social bots more like human users before they start to infiltrate Sina Microblog, every social bot is randomly assigned to 4–8 other social bots as initial followers and they will post at least 10 microblogs consecutively within 5 days before infiltration actions start.

### Gender

**Figure 8:**
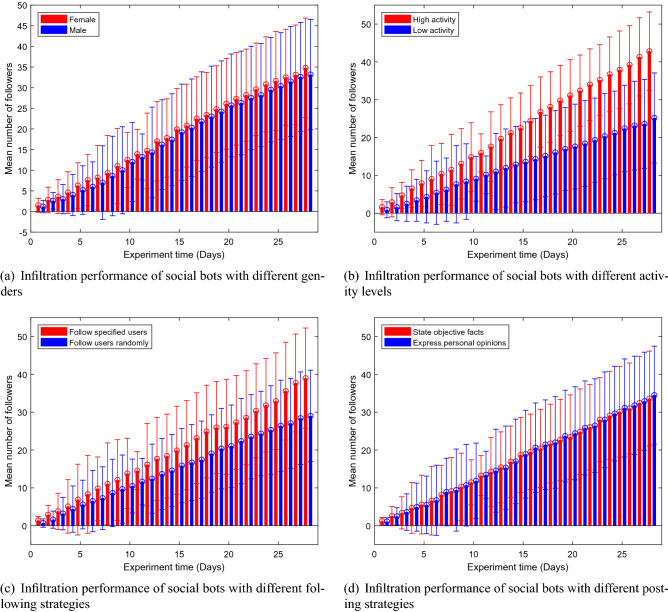
Infiltration performance of social bots with different genders, activity levels, following strategies and posting strategies. The bars represent the mean values and the error bars denote the standard deviation.

**Figure 9:**
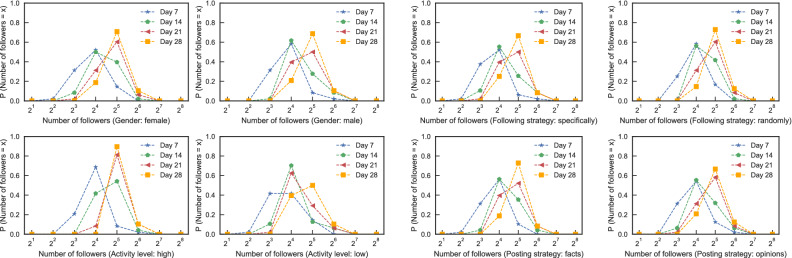
The probability distribution of the number of social bots’ followers under different strategies on days 7, 14, 21 and 28. In order to make connecting lines smoother, $P( number\, of\, followers =2^i)$ represents the sum of the probabilities where the number of followers belongs to $[1.5\cdot 2^{i-1}, 1.5\cdot 2^{i+1})$.

We first analyzed the impact of social bots’ gender on the infiltration performance in our experiment. Figure 8a shows the changes in the mean number of followers of female and male social bots respectively over time during the first four weeks of experiments. The bars represent the mean values and the error bars denote the standard deviation. The mean number of followers is calculated as Eq. (3), where *M* is the mean number of followers, *S* is the target social bots set, *n* is the number of social bots in this set and $s_{fi}$ is the number of followers of the *i*th social bot.

3 $$\begin{aligned} M=\frac{\sum \nolimits ^{n}_{i=1} s_{f1} +s_{f2} +\cdots s_{fi}+\cdots +s_{fn}}{n},s\in S \end{aligned}$$

It can be seen from Fig. 8a that the mean number of followers of both female and male social bots are steadily increasing. In general, the infiltration performance of female ones is slightly better than that of male ones, but the user’s gender setting does not have a significant effect on the infiltration performance of social bots. This is because the majority of users do not value the attribute of gender in profile for they often setting it as fake. Compared to this, the profile photo of a real woman can better indicate that this user is female.

### Activity level

According to the frequency of social bots posting microblogs and following users, we defined the activity frequency of social bots as high and low activity levels(“Infiltration strategies of social bots”).

Figure 8b shows the mean number of followers of social bots with high and low activity levels over time. It can be seen that the mean number of followers obtained by high-activity social bots is much higher than that of low-activity social bots. This is because the more active the social bot is, the more likely it is to be seen by other users, so it is easier to get more followers. This proves that the infiltration strategies in this paper have achieved a good balance between security and activity. They are effective and can keep social bots from being detected by Sina Microblog defense mechanism while maintaining high activity (Fig. 9).

### Profile photo

In this article, social bots’ profile photos were set to real human photos and unreal human photos with a ratio of 1:1.

**Figure 10:**
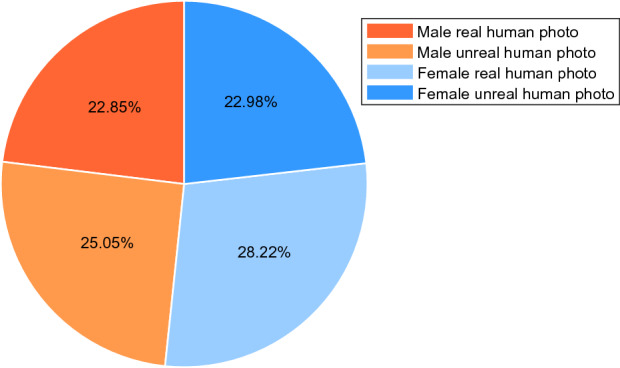
The proportion of social bots’ followers using different kinds of profile photos.

Figure 10 shows the proportion of social bots’ followers using different profile photo setting strategies.As can be seen from Fig. 10, the proportions of valid followers obtained by social bots with real human profile photos and those with unreal human profile photos are 51.08% and 48.92%, respectively. Even though followers obtained by social bots with real human profile photos is slightly more, the profile photo setting has no obvious effect on the infiltration performance as a whole. Combined with the attribute of gender, female social bots using real women photos have 2–6% more followers than others. This indicates that females are more popular in Sina Microblog OSN, which is consistent with the research results of Bilge et al.^65^.

### Following strategy

We divided all social bots into 3 groups according to different interested topics. In each group, half of social bots were set to follow users randomly and the other half were set to only follow users who posted microblogs on the same interested topic. Figure 8c shows the mean number of followers of social bots using different following strategies over 4-week experimental time. As can be seen from the figure, social bots who followed specific groups are more likely to get more followers than those who randomly followed users, which is the same with Freitas et al.^29^.

### Posting strategy

This subsection aims to research the best microblog posting strategy. Figure 8d shows the mean number of followers of social bots with different kinds of microblogs: subjective ones—personal opinion or objective ones—news facts. On the whole, there is no difference between them on the infiltration performance of social bots. This is because the opinions and news we post were all about the same topic and the same topic itself has already resonated to some extent.

Figure 9 further shows the probability distribution of the number of followers in Fig. 8 over time. We found that these distributions are extremely close to the normal distribution and the center moves forward with time. This reveals that although the number of followers under different strategies is constantly changing and increasing, its probability distribution still maintains a normal distribution.

### Quantifying the influence of followers

In Sina Microblog, users’ microblogs will be automatically pushed to their followers due to its message transmission mechanism, resulting in that the quality of followers further determines how much influence social bots can have. Hence, quantifying the followers of social bots is of great importance when researching social bots’ influence on other users. The user influence of Sina Microblog, which can be used to quantify the followers, measures the impact of a user on the overall information propagation of this platform. So, we use the total influence of each social bots’ followers as the quality of its followers. According to Ref.^66^, a user’s influence is usually determined by the influence of microblogs, the proliferation influence of message propagation, and the activity level. We quantified the influence of the followers from these three aspects based on Ref.^66^ as well.

(a) The influence of microblogs

According to Ref.^66^, the influence of microblogs on time sequence is a cumulative process, so the influence of a user’s microblogs is taken by the means of the influence of all microblogs posted by the user, calculated as Eq. (4). The $mcrb_Influence(j)$ represents the influence of the *j*th microblog of follower $f_i$. And considering followers can only spread the microblogs posted by social bots during their survival, *N* represents the number of microblogs posted by follower $f_i$ during the 4-week experimental time.

4 $$\begin{aligned} MicroblogIf(f_i)=\frac{\sum \nolimits _{j=1}^n{mcrbInfluence(j)}}{N} \end{aligned}$$

A microblog can be commented, liked or forwarded by other users, so its influence is defined as follows:

5 $$\begin{aligned} mcrbInfluence(j)=CoNum(j)+LikeNum(j)+ForNum(j), \end{aligned}$$

where *CoNum*(*j*) is the number of comments, *LikeNum*(*j*) is the number of liking, and *ForNum*(*j*) is the number of forwarding of microblog *j* by other users.

(b) The proliferation influence of message propagation

In Ref.^66^, the influence of message propagation is divided into two aspects: the direct influence and the indirect influence. Given that there has been a gap in the dissemination of information between the followers’ followers and social bots, we only consider the direct one. That is defined as

6 $$\begin{aligned} ProliferationIf(f_i)=mcrbNum_{f_i}*faNum_{f_i}, \end{aligned}$$

where $mcrbNum_{f_i}$ is the number of microblogs posted by user $f_i$ and $faNum_{f_i}$ is the number of followers of user $f_i$.

(c) The activity level

The follower’s activity level reflects how active he or she is in Sina Microblog. It is defined as the mean number of microblogs posted per day. It is calculated as

7 $$\begin{aligned} ActiveIf(f_i)=\frac{\sum \nolimits _{k=1}^D{mcrbNum_k}}{D}, \end{aligned}$$

where $mcrbNum_k$ is the number of microblogs posted by follower $f_i$ at the *k*th day and *D* is the total number of experimental days, set to 28.

(d) Quantifying the influence of followers

As there is a big difference between the number of followers, comments, liking and forwarding, these data must be normalized before calculating the influence of followers to make them at the same magnitude. This paper normalizes these indicators according to Eq. (8), where *Normal*(*j*) represents the normalized result of the *j*th indicator and ${\overline{x}}_{j}$, $x_{max,j}$, $x_{min,j}$ represent the average value, maximum value and minimum value of the *j*th indicator.

8 $$\begin{aligned} Normal(j) =0.5*\left( 1+\frac{x_j -{\overline{x}}_{j}}{x_{max,j} -x_{min,j}}\right) \end{aligned}$$

Then Referring to Ref.^2^, the *i*th follower $f_i$’s influence, is defined as

9 $$\begin{aligned} Influence(f_i)=\lambda _1MicroblogIf(f_i)+\lambda _2ProliferationIf(f_i)+\lambda _3ActiveIf(f_i), \end{aligned}$$

where $\lambda _1$, $\lambda _2$, and $\lambda _3$ are weight parameters and are set to $\lambda _1=0.34$, $\lambda _2=0.53$, and $\lambda _3=0.13$ respectively. After quantifying the influence of each follower, we can calculate the average follower influence of social bots in each strategy. For *s*th social bot $s_s$ , the total influence of its followers, $SocialbotIf(s_s)$, is defined as

10 $$\begin{aligned} SocialbotIf(s_s)=\sum \limits _{i=1}^F{Influence(f_i)}, \end{aligned}$$

where *F* represents the number of *m*th social bot’s followers. And for each strategy, the average follower influence of social bots is gotten as follows:

11 $$\begin{aligned} StrategyIf(strategy_m)=\frac{\sum \nolimits _{s=1}^S{SocialbotIf(s_s)}}{S}. \end{aligned}$$

$StrategyIf(strategy_m)$ represents the average follower influence of social bots in *m*th strategy, and *S* represents the number of social bots in *m*th strategy.

**Table 6:** The average follower influence of one social bots under different strategies.

Strategy	Setting	The average follower influence of social bots
Gender	Female	10.0729
	Male	10.2937
Activity level	High	12.2426
	Low	8.1240
Profile photo	Real human photo	10.3464
	Unreal human photo	10.0202
Following strategy	Follow specified users	9.7199
	Follow users randomly	10.6467
Posting strategy	State objective facts	10.1794
	Express personal opinions	10.1873

The $StrategyIf(\cdot )$ of each strategy is shown in Table 6. As we can see from the table, the average follower influence of social bots with different genders, profile photos and posting strategies is actually very close, and the average follower influence of social bots with high activity is significantly higher than that of other strategies and low activity. This ravels that gender, the type of profile photo and the different posting strategies make little difference in expanding the influence of social bots, while high activity can help social bots gain more influence quickly. However, as for the following strategy, although following specific groups can gain more followers, the quality of these followers is not as high as that obtained by randomly following users. This is because there are some zombie users or other social bots in the specified groups we followed, and even though they followed back all requests, none of them will have any value.

### Performance analysis of interactive actions

The five subsections analyze the infiltration performance of five infiltration strategies and this chapter further researched the infiltration performance of the four common interaction behaviors including following, commenting, forwarding and liking.

Firstly, we selected four social bots from these three topic groups respectively and the numbers of their followers are close. The four social bots in the same topic group only performed following, commenting, forwarding and liking actions respectively during the experimental time. Then the attributes of these 12 social bots that might affect the infiltration performance were set to the same, like ages profile photos and so on. After that each social bot performed the corresponding interactive action 30 times a day. Furthermore, this experiment was not started until no new followers were gained by these social bots which lasted for 5 days.

The strategy for selecting target users in this section is similar to the following strategy in “Infiltration strategies of social bots”, but with stricter restrictions: the target users must be active within 3 days in Sina Microblog. (If the user has performed related actions such as commenting, posting and following within 3 days, then the user is active.) So we captured the microblogs posted by V users on each topic on the day and the relevant users who commented, forwarded and liked these microblogs must be users who have been active within 3 days. This section took them as target users.

**Figure 11:**
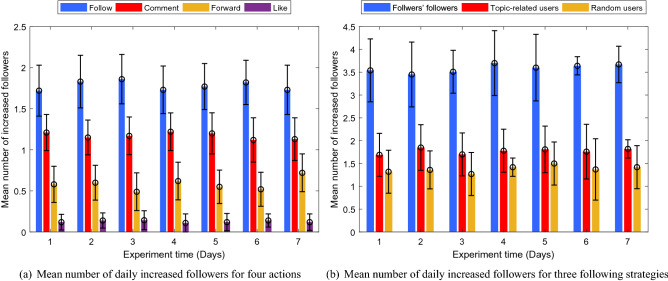
Mean number of daily increased followers for four actions and three following strategies.

When performing a following action, social bots only follow target users interested in the same topic; when performing a liking action, social bots only give likes to the latest microblogs of target users who interested in the same topic; when performing a forwarding action, social bots select one from the captured topic-related microblogs and @*the target user*; when performing a commenting action, social bots select one microblog from the forwarded microblogs to comment. The average daily growth of followers of these social bots is as shown in Fig. 11a.

It can be seen from these two graphs that each type of action will lead to a steady increase in the number of followers, but the following action can best promote the social bots to acquire followers and also is the greatest promotion of influence acquisition, followed by the commenting action and the forwarding action. However, the liking action has no significant effect on influence acquisition.

Because the following action has better infiltration performance than other actions in the interactive actions of social bots, so we further research on following strategies.

Similarly, we selected 3 social bots, the number of whose followers were close, from 3 topic groups and adjusted their attributes that might affect the infiltration performance to the same. Then let each social bot perform 30 following actions per day. Furthermore, this experiment was not started until no new followers were gained by these social bots which lasted for 5 days. Three different following strategies was studied: (1) randomly following target users that have not yet been followed; (2) following users who are interested in the same topic of social bots; (3) following followers of the social bot’s own followers. This experiment lasted a week in Sina Microblog. The average daily growth of followers of these social bots is showed in Fig. 11b.

As can be seen from Fig. 11b, although the number of followers has been steadily increasing under all following strategies, the number of followers obtained by the third following strategy is almost double that of the other two strategies. So following the followers of followers is better than the other two strategies for infiltration performance. This proves that the homophily which is the tendency of individuals to associate and bond with similar others in social networks makes social bots in Sina Microblog OSN more influential, which is the same with social bots in Twitter^28^.

## Conclusion and future work

In this paper, we built 96 social bots through reversely analyzing the login process and network packages from Sina Microblog to explore the influence of different characteristics of social bots. 5546 followers social bots gotten and their 100% survival rate in the experimental time not only indicate the effectiveness of our infiltration strategies but also implicate the vulnerability of Sina Microblog defense mechanism. We believe that our findings have a number of implications for designers of spammer defense mechanisms in OSNs. On the other hand, these social bots can be a weapon against the malicious interpretation of current events in Sina Microblog as well. In short, we can draw the following conclusions based on our studies.

Firstly, we proposed a fusion strategy which combines simulated login and visitor cookies to crawl Sina Microblog data automatically through cracking login process and analyzing related communication protocols. The experimental result shows that on the premise that social bots will not be detected, using the fusion strategy to collect data is much faster than using official APIs. And we can even get users’ social relationships for which Sina Microblog does not provide the API.

Secondly, we set up five infiltration strategies and two comparison experiments about four different interactive actions to analyze the main factors that may influence social bots’ influence. In the process of constructing social bots, we designed a set of guidelines to automate the initialization of social bots. We gave them human-like profiles and create a set of action commands to control their daily activities. The experimental results prove that the gender, the type of profile photos and subjectivity or objectivity of microblogs have little effect on the influence of social bots, while the activity level, the following strategy and the type of interactive actions can make a big difference for social bots.

Based on the experimental analysis in “Performance analysis of Interactive actions the five subsections analyze the infiltration performance of”, we conclude that if social bots want to acquire a certain number of followers as soon as possible, the following strategies are recommended:

- Gender has little impact on social bots but the female gender and corresponding attribute settings are still recommended compared to the male gender. Because on average, female social bots are more popular than male social bots.
- The high activity level is recommended. Because it gives social bots more opportunity to be seen by others.
- Following is the fastest way to gain followers among four interactive actions, followed by commenting. So these two actions are most recommended. However, it should be noted that zombie users or other social bots should be avoided as much as possible when performing following actions because these users have little value.
- Make good use of homophyly theory because it has more impact on the infiltration scale. You can do it from the following suggestions: select users who have the same interest as target users and try to infiltrate the followers of followers.

Although this work contributes to the analysis of social bots and defense mechanism of OSN, there are limitations to be considered and areas of improvement in future work. The scale of the constructed social bot dataset can be enriched from different dimensions. In this paper, five factors which intuitively affect how successfully a social bot infiltrate OSN were studied. However, other factors, such as the polarity and content of speech, may also affect the influence of social bots. So we take it as a potential future work. At the same time, the number and active time of social bots can also be further extended to find more general patterns.
